# Fluorinated Montmorillonite and 3YSZ as the Inorganic Fillers in Fluoride-Releasing and Rechargeable Dental Composition Resin

**DOI:** 10.3390/polym12010223

**Published:** 2020-01-16

**Authors:** Keng-Yuan Li, Cheng-Chia Tsai, Tzu-Chieh Lin, Yin-Lin Wang, Feng-Huei Lin, Chun-Pin Lin

**Affiliations:** 1 Institute of Biomedical Engineering, National Taiwan University, No.49, Fanglan Rd., Da’an Dist., Taipei 10672, Taiwan; plee0306@hotmail.com (K.-Y.L.); jacklin7412@gmail.com (T.-C.L.); 2 Department of Neurosurgery, Mackay Memorial Hospital, No.92, Sec. 2, Zhongshan N. Rd., Zhongshan Dist., Taipei 10449, Taiwan; angle@mmh.org.tw; 3 Graduate Institute of Clinical Dentistry, School of Dentistry, National Taiwan University, No.1, Sec. 1, Ren’ai Rd., Zhongzheng Dist., Taipei 10051, Taiwan; wil1019@ntu.edu.tw; 4 National Taiwan University Hospital, College of Medicine, National Taiwan University, No.1, Changde St., Zhongzheng Dist., Taipei 10048, Taiwan; 5 Institute of Biomedical Engineering and Nanomedicine, National Health Research Institutes, No.35, Keyan Rd., Zhunan Township, Miaoli County 35053, Taiwan

**Keywords:** dental caries, fluorides, composite resins, Bis-GMA, bentonite, zirconium

## Abstract

Dental caries (tooth decay) is the most frequent oral disease in humans. Filling cavities with a dental restorative material is the most common treatment, and glass ionomer cements are the main fluoride ion release restorative materials. The goal of this study was to develop a restorative compound with superior fluoride ion release and recharge abilities. Previously developed fluorinated bentolite and hydrophobized 3YSZ were used as two different inorganic fillers mixed in a bisphenol A-glycidyl methacrylate (Bis-GMA) matrix. XRD, FTIR, and TGA were used to determine the hydrophobic modification of these two inorganic fillers. In mechanical tests, including diameter tensile strength, flexural strength, and wear resistance, the developed composite resin was significantly superior to the commercial control. A WST-1 assay was used to confirm that the material displayed good biocompatibility. Furthermore, the simulation of the oral environment confirmed that the composite resin had good fluoride ion release and reloading abilities. Thus, the composite resin developed in this study may reduce secondary caries and provide a new choice for future clinical treatments.

## 1. Introduction

Dental caries, commonly called tooth decay, is one of the most frequent chronic diseases worldwide [1]. According to the WHO, nearly 100% of people have dental caries [2]. Oral health is at the basis of general health of patients, and poor oral health can affect their systemic health conditions [3]. Importantly, tooth decay can be prevented by maintaining a constant low level of fluoride in the oral cavity [4]. Fluoride is considered the most effective treatment against dental carries, as fluoride ions have been shown to inhibit acid production from bacteria, reduce demineralization and promote remineralization of enamel and dentin [5,6,7]. Recent studies have indicated that fluoride-containing restorative materials inhibit demineralization and the development of caries [8,9], as well as strengthening neighboring tooth enamel and dentin [7,8], thereby decreasing chances of tooth decay.

Although dental filling materials have been evolving over the past years, few materials meet clinical needs. For example, gold and silver are too soft, the color of amalgam alloy is black and has no chemical bonding with dentin, the solubility and permeability of silicate cement are too high [10,11,12], and amine peroxide in PMMA leads to color changes and the wear resistance is insufficient [13]. The most common filling materials currently on the market are glass ionomer cements (GICs) and light-cured composite resins (LCCs). GICs are made from polyacrylic acid solutions and silicon aluminum fluoride glass powder (SiO_2_-Al_2_O_3_-CaF_2_-AlPO_4_-Na_3_AlF_6_); the advantage of these materials is the ability to form chemical bonds with enamel and dentin [14,15,16]. Compared to composite resins, GIC materials also exhibit higher fluoride ion release amount [17]. Unfortunately, these materials have many clinical usage problems, such as poor handling properties and high solubility in an early setting stage. At the same time, mechanical properties of GIC materials are not sufficient for use in molar restoratives [18,19]. An LCC, a composite of bisphenol A-glycidyl methacrylate (Bis-GMA), was synthesized by Dr. Bowen in 1962 [20,21]. When an LCC is combined with inorganic fillers, it has good syringe flow and can be set by UV light; furthermore, it is not soluble in the oral cavity and possesses remarkable mechanical properties including excellent hardness, wear resistance, and compression strength [22]. Although many studies have shown that antibacterial agents, such as chitosan, fluorine, and chlorine, can be added to composite resins [3], nearly all LCC commercial products have poor fluoride release properties [23,24].

Bentonite is also called montmorillonite (MMT). It is a layered smectic clay formed by silicon oxide with a tetrahedral structure and aluminum hydroxide with an octahedral structure. Previous studies have confirmed that MMT can be made hydrophobic by treatment with N-methylformamide (NMF) and acrylamide (AAm); treatment with hydrofluoric acid to form fluorinated MMT provides fluoride ion release and recharge abilities. While these properties make it a good inorganic filler in the composite resin, the mechanical properties are not sufficient for use as a restorative material, but only as a sealant [25].

The use of two or more inorganic substances of different sizes as fillers in a composite resin can improve the mechanical properties of the composite resin and create a smooth surface [26]. Therefore, 3YSZ was used as a second inorganic filler in this study. 3YSZ is a 3% mol yttria-stabilized zirconia [27]. The nanometer-grade 3YSZ has good optical properties and mechanical strength [28,29,30]. In order to have a good bonding between 3YSZ and the polymer matrix, silane treatment is needed. The silane agent we used was (3-Mercaptopropyl)trimethoxysilane (MPTMS), as its methacrylate functional group can bind with the polymer matrix. Further, MPTMS is the most commonly used silane in dental applications, because its methacrylate functional group matches most dental resins [31].

This study aimed to use a mixture of fluorinated MMT and hydrophobic 3YSZ to develop a new LCC dental restorative material with good fluoride release and recharge abilities, strong mechanical properties, and biocompatibility for clinical applications.

## 2. Materials and Methods

### 2.1. Material Preparation

#### 2.1.1. Preparation of the NaF-Intercalated Fluorinated Montmorillonite (FMMT/AAm-NaF) Filler

The FMMT filler production method was previously described [25]. Briefly, HF (Sigma-Aldrich, St. Louis, MO, USA) and H_2_SO_4_ (Sigma-Aldrich, St. Louis, MO, USA) were used to convert some of OH^−^ groups in MMT (Sigma-Aldrich, St. Louis, MO, USA) into F^−^ groups to change MMT to FMMT with fluoride ion release and recharge capabilities. NMF (Sigma-Aldrich, St. Louis, MO, USA) was used to insert the 001 plane spacing of the FMMT and was then further replaced by AAm (Alfa Aesar, Ward Hill, MA, USA) to expand the plane spacing again; this changed FMMT from hydrophilic to hydrophobic. Finally, NaF (Sigma-Aldrich, St. Louis, MO, USA) was added to the material to further increase the fluoride ion concentration. In this study, the modified MMT filler was abbreviated as FMMT/AAm-NaF.

#### 2.1.2. 3YSZ Silane Treatment with MPTMS 

The 3YSZ material (1 g; provided by the Ceramic Composite Lab, National Taiwan University, Taipei, Taiwan) was well-mixed with 2 mL MPTMS (Sigma-Aldrich, St. Louis, MO, USA) by magnetic stirring at 25 °C for 24 h. The mixture was centrifuged (6000 rpm, 10 min), and the supernatant was discarded. The resulting product was dried in a 60 °C vacuum oven to obtain the 3YSZ/MPTMS powder. In this study, the modified 3YSZ filler was abbreviated as 3YSZ/MPTMS.

#### 2.1.3. Preparation of the Composite Resin

A fluoride-releasing composite resin (FCR) was mixed with the FMMT/AAm-NaF, 3YSZ/MPTMS, and polymer matrix. The polymer matrix was mixed by Bis-GMA, triethylene glycol dimethacrylate (TEGDMA), N, N-dimethyl-p-toluidine (DMPT), and camphorquinone at a weight ratio of 159:39:1:1 (all materials purchased from Sigma-Aldrich, St. Louis, MO, USA). The FMMT/AAm-NaF and 3YSZ/MPTMS acted as inorganic fillers in the FCR prepared using a light-curable polymer matrix at 20 wt % FMMT/AAm-NaF and 20 wt % 3YSZ/MPTMS in the composite resin. Polymerization was performed by using blue light with a wavelength range of 460–510 nm (Litex 696, Dentamerica, City of Industry, CA, USA).

### 2.2. Materials Analysis and Characterization: XRD, FTIR, TGA, and Particle Size

XRD was used to confirm the 3YSZ crystal structure. The change in the plane spacing of the (001) plane in the FMMT/AAm-NaF was also examined. The 2θ angle of the MMT (001) plane was transformed using Bragg’s law to evaluate the change in plane spacing. The sample powder was mounted on a holder of an X-ray diffractometer (Miniflex, Rigaku, Tokyo, Japan) under Cu KαI radiation (λ = 0.15406 nm). The scanning range of MMT was from 2° to 8°, the scanning range of 3YSZ was from 25° to 65°, and the scanning rate was 1°/min. Characteristic peaks were used to identify the crystal structure.

FTIR spectroscopy was used to compare the spectra of the 3YSZ/MPTMS and FMMT/AAm-NaF with those of pure MPTMS and AAm to confirm successful modification.

TGA was used to observe the change in sample weight at different temperatures. The temperature of the sample was increased from 100 to 700 °C at a rate of 5 °C/min to compare the percentage weight of the FMMT/AAm-NaF and 3YSZ/MPTMS at 700 °C, in order to determine the polymer contents in these two inorganic fillers. We collected data from 100 °C to remove the effects of moisture and set the weight at 100 °C as 100%.

In the particle size measurement, 0.1 g of the FMMT/AAm-NaF and 3YSZ/MPTMS was dispersed in 2 mL DI water. A light-scattering instrument (Zetasizer nano, Malvern Co., Malvern, Worcs, UK) was used to measure the particle size.

### 2.3. Mechanical Analysis: Curing Depth, Hardness, Diametral Tensile Strength, Flexural Strength, and Wear Resistance

The light penetration of the FCR was measured by curing depth tests. The curing depth of the light-curing composite resin should be at least 1.5 mm according to the ISO 4049 standard [32]. The sample was prepared with 4 mm in diameter and 6 mm in depth by a stainless-steel mold. After filling the material, blue light (wavelength: 460–510 nm; Litex 696) was used unidirectionally, and the curing depth was measured after demolding.

A stainless-steel mold with 6 mm in diameter and 3 mm in height was used for FCR hardness tests. After irradiating blue light from both sides and demolding, the sample surfaces were wet-polished with 1000-grit silicon carbide paper. An HMV-2 tester (Shimadzu, Tokyo, Japan) was used at a 0.05 kg load and a 30 s dwell time. We measured six points on each sample and averaged the hardness of these six points as the hardness of the sample. Six samples were measured using the same conditions and parameters, and the mean and standard deviation were calculated.

Diameter tensile strengths were measured according to the New American Dental Association Specification No. 27 [33]. Samples were prepared in a stainless-steel mold with 3 mm in height and 6 mm in diameter and polymerized using the method described above. Samples containing air bubbles or defects visible to the naked eye were excluded. Silicon carbide paper was used to remove flash; this method was also used for subsequent flexural strength tests and wear resistance tests. The samples were mounted between disks of a universal testing machine (HDX, Instron, Norwood, MA, USA) and measured with a crosshead speed of 0.5 mm/min. The diametral tensile strength was calculated by Equation (1) [34]:

(1) $$\sigma_{t}=\frac{2P}{\pi \mathrm{DT}},$$

where σt is the tensile stress, P is the load at fracture, π is ratio of the circumference of a circle to its diameter, D is the diameter, and T is the thickness.

Flexural strength tests were performed according to the ISO 4049 standard [35]. The cross-sectional area of the samples was 4 mm^2^ (sample size: length, 24 mm; height, 2 mm; width, 2 mm). After cured and demolded, samples were placed on a three-point bending test device with a 20 mm distance. The universal testing machine crosshead speed was also 0.5 mm/min. The flexural strength (α) was calculated by the following equation [36]:

(2) $$\alpha =\frac{3\mathrm{FL}}{2{\mathrm{bh}}^{2}},$$

where F is the load (kN), L is the span length (cm), h is the sample thickness (cm), and b is the sample width (cm).

The wear resistance test protocol we followed was described in a previous study [25]. An acrylic mold (3 mm in diameter and 20 mm in height) was used. Blue light irradiation occurred around the acrylic mold for 60 s to cure the composite resin. A tribometer (UMT-2, CETR, Campbell, CA, USA) with a 10 N loading on a 400-grit silicon carbide grinding paper was used with a rotational speed of 100 rpm for 500 cycles. The wear resistance was calculated by dividing the remaining weight by the original weight.

### 2.4. Oral Environment Simulation

#### 2.4.1. Fluoride Release Measurement

For fluoride release and recharge ability tests, we used a commercial product Fuji IXGP (GC, Bunkyo, Tokyo, Japan) as the control. Six samples (6 mm in diameter and 2 mm in height) were prepared and polymerized as described above. The samples were placed in 5 mL of DI water, which was renewed every day for 14 days. The fluoride concentration in the daily renewed DI water was measured by ion chromatography (883, Metrohm, Herisau, AR, Switzerland), and the amount per square centimeter was calculated by Equation (3):

(3) $$\frac{\mu \mathrm{gF}}{{\mathrm{cm}}^{2}}=\mathrm{ppm}\;F\left(\frac{\mu \mathrm{gF}}{\mathrm{mL}}\right)\times \mathrm{mL}\left(\mathrm{volume}\;\mathrm{of}\;\mathrm{medium}\right)\times \frac{1}{\mathrm{surface}\;\mathrm{area}\;\mathrm{of}\;\mathrm{specimen}\;\left({\mathrm{cm}}^{2}\right)}$$

#### 2.4.2. Fluoride Recharge Measurement

After 14 days of fluoride release, residual fluoride ions on the surface of the samples were removed by sonication (three times in 15 mL DI water for 10 min). Subsequently, the samples were placed in a 0.2% NaF solution (5 mL) at pH 7 for 1 min [37] to recharge fluoride ions to the samples. Then, the samples were removed, air-dried and soaked in DI water (5 mL) to repeat the fluoride ion release process over 1 week. The fluoride released for 7 days was calculated by using the equation mentioned above.

### 2.5. Biocompatibility

#### WST-1 Assay

To determine the biocompatibility, the composite resin was tested using the WST-1 assay with 3T3 cells following the ISO 10993-5 standard [38], with an extraction solution prepared according to the ISO 10993-12 standard [39]. The FCR was crushed to powder, added to Dulbecco’s modified Eagle Medium (DMEM) at a concentration of 0.2 g/mL and incubated at 37 °C in 5% CO_2_ atmosphere for 3 days to produce the extraction solution. The 3T3 cells were seeded at a density of 1 × 10^4^ cells/well in a 96-well plate and incubated in a 5% CO_2_ atmosphere at 37 °C for 1 day. Then, the cell culture medium (DMEM) was replaced with the extraction solution, and the cells were incubated for 3 days. Cells kept in DMEM were used as the control group, and those, to which 1% Triton X-100 was added, served as the positive control group. After incubation, the plates were placed in an ELISA reader (Epoch 2, BioTek, Highland Park, VT, USA) set to record the absorbance at 450 nm (with a reference filter at 600 nm) associated with Formazan formation.

### 2.6. Statistical Analysis

Statistical analysis was performed from data collected and presented as the mean ± standard deviation (SD). Differences were considered statistically significant, when the *p*-value was less than 0.005. * represents *p* < 0.05, ** indicates *p* < 0.005, and *** denotes *p* < 0.001.

## 3. Results

### 3.1. Material Characterization: XRD, FTIR, TGA, and Particle Size

#### 3.1.1. XRD

**Figure 1:**
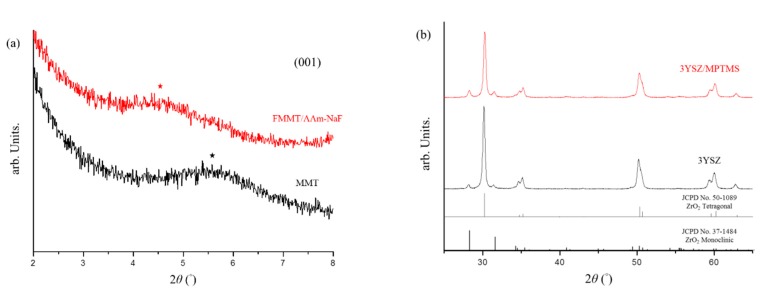
XRD analysis of montmorillonite (MMT) and the NaF-intercalated fluorinated montmorillonite (FMMT/AAm-NaF): (**a**) XRD patterns of MMT and the FMMT/AAm-NaF from 2° to 8°. The (001) plane angle is marked with a star; (**b**) XRD patterns of 3YSZ and the 3YSZ/MPTMS from 25° to 65° compared with the underlined references of the Joint Committee on Powder Diffraction Standards (JCPDS) card No. 50-1089 for ZrO_2_ tetragonal and No. 37-1484 for ZrO_2_ monoclinic.

XRD was used to determine the 001 plane diffraction angle of MMT after a series of modifications. The 2θ angle changed from 5.59° (MMT) to 4.53° (FMMT/AAm-NaF) (Figure 1a). The provided 3YSZ was compared with the hydrophobized 3YSZ/MPTMS (Figure 1b), and the data indicated that the crystal structure of 3YSZ did not change after the hydrophobic treatment.

#### 3.1.2. FTIR

**Figure 2:**
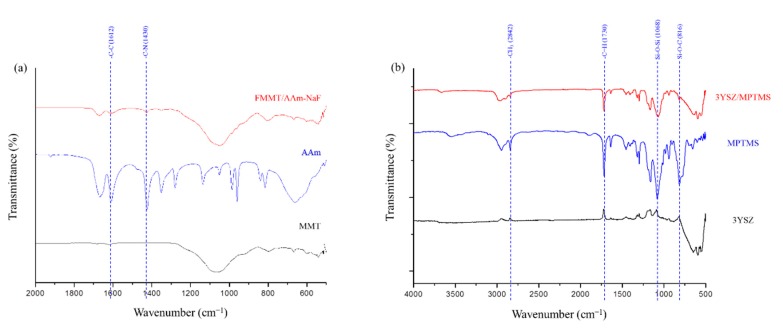
FTIR spectra analysis of the prepared composites: (**a**) FTIR spectra (500–2000 cm^−1^) of MMT, AAm, and the FMMT/AAm-NaF; (**b**) FTIR spectra (500–4000 cm^−1^) of 3YSZ, MPTMS, and the 3YSZ/MPTMS.

FTIR was used to confirm the modification of the inorganic fillers, including the FMMT/AAm-NaF and 3YSZ/MPTMS, contained in the polymers. The patterns were compared with those of pure AAm (Figure 2a) and MPTMS (Figure 2b). The spectrum of AAm (Figure 2a) displayed the AAm characteristic bands at 1612 cm^−1^ (C–C) and 1430 cm^−1^ (C–N), which were not observed in the MMT spectrum but were present in the spectrum of the FMMT/AAm-NaF. In Figure 2b, the bands at 2842 cm^−1^ (–CH_3_) and 14,730 cm^−1^ (C=H) were characteristics of MPTMS and were not observed in the spectrum of 3YSZ. In addition, the 1068 cm^−1^ (Si–O–Si) and 816 cm^−1^ (Si–O–C) absorption bands in the MPTMS spectrum became less pronounced after the reaction with 3YSZ.

#### 3.1.3. TGA

**Figure 3:**
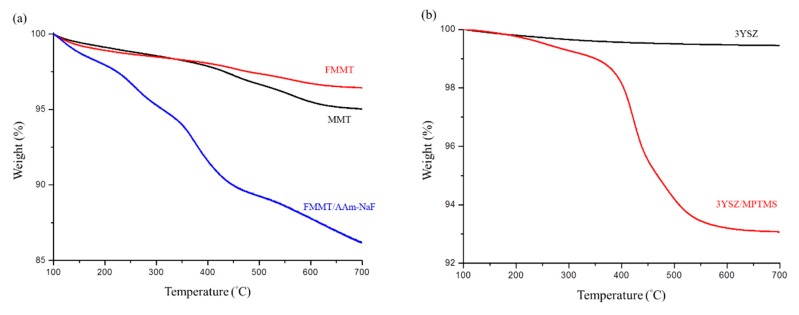
Thermogravimetric curve analysis: (**a**) TGA patterns of MMT, FMMT, and the FMMT/AAm-NaF between 100 and 700 °C; (**b**) TGA patterns of 3YSZ and the 3YSZ/MPTMS between 100 and 700 °C.

The thermogravimetric curve of the FMMT/AAm-NaF indicated that ~10.66 wt % of AAm was present in the FMMT (Figure 3a). The MPTMS content of the coated 3YSZ was ~6.39 wt % (Figure 3b). The polymers were not immediately decomposed by being heated to the boiling points owing to the shielding effect of the FMMT.

#### 3.1.4. Particle Size

**Figure 4:**
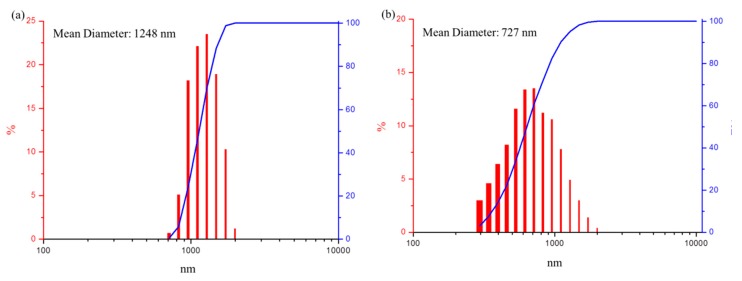
Particle size and cumulative size distribution analysis: (**a**) particle size distribution (red columns) and cumulative size distribution (blue line) of the FMMT/AAm-NaF; (**b**) particle size distribution (red columns) and cumulative size distribution (blue line) of the 3YSZ/MPTMS.

The particle size of the FMMT/AAm-NaF was determined to be ~1248 nm (Figure 4a). There was almost no aggregation in the 3YSZ/MPTMS during the drying process after the hydrophobization treatment. 3YSZ had a small particle size, with an average diameter of ~727 nm (Figure 4b).

### 3.2. Mechanical Analysis: Curing Depth, Hardness, Diametral Tensile Strength, Flexural Strength, and Wear Resistance

#### 3.2.1. Curing Depth

**Figure 5:**
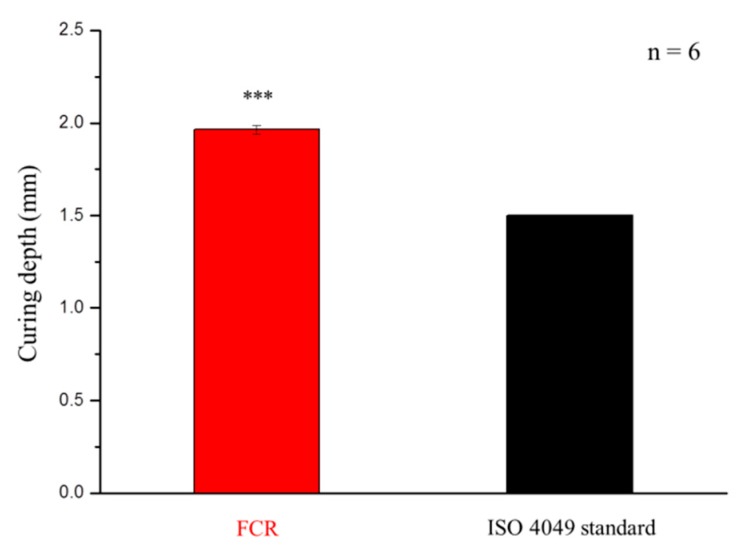
Curing depths of the developed fluoride-releasing composite resin (FCR) and stated in the ISO 4049 standard. *** denotes *p* < 0.001.

As shown in Figure 5, the curing depth of the FCR was higher than that shown in the ISO 4049 standard (1.5 mm).

#### 3.2.2. Hardness, Diametral Tensile Strength, Flexural Strength, and Wear Resistance

**Figure 6:**
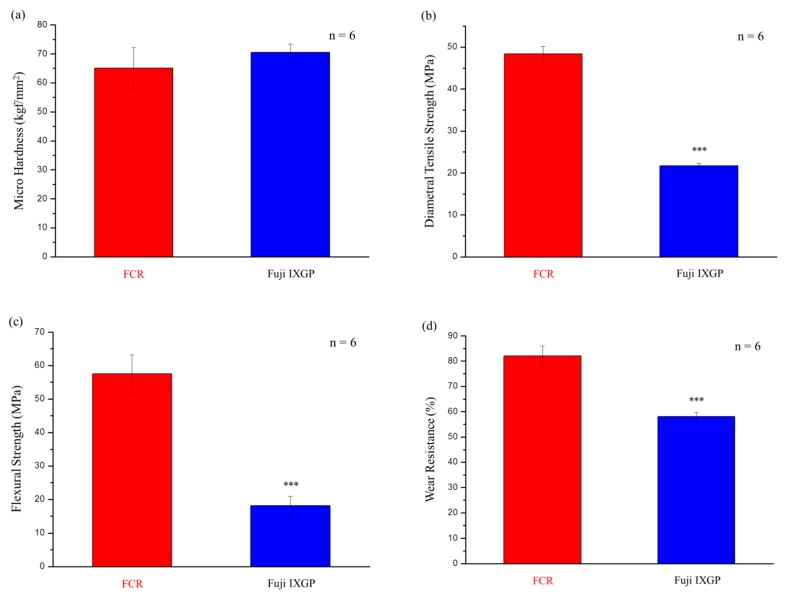
Mechanical properties of the FCR and the commercial product Fuji IXGP: (**a**) microhardness; (**b**) diametral tensile strength; (**c**) flexural strength; (**d**) wear resistance. *** denotes *p* < 0.001.

No statistically significant difference was observed between the commercial control (Fuji IXGP) and the FCR in hardness tests (*p* = 0.055, Figure 6a). However, the values of the FCR in diametral tensile strength (*p* = 4.99 × 10^−12^, Figure 6b), flexural strength (*p* = 1.46 × 10^−8^, Figure 6c), and wear resistance (*p* = 4.41 × 10^−8^, Figure 6d) were higher than those of the control group.

### 3.3. Oral Environment Simulation.

#### 3.3.1. Fluoride Release Measurement

**Figure 7:**
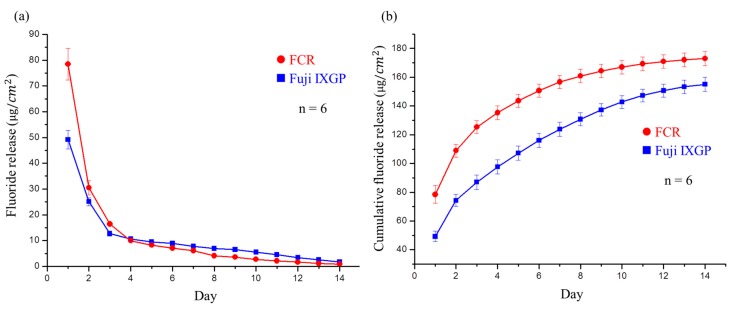
Fluoride release analysis of the developed FCR and commercial Fuji IXGP: (**a**) fluoride release per cm^2^ of the FCR and Fuji IXGP each day for two weeks. No significant difference was observed on day 4; (**b**) cumulative fluoride release of the FCR and Fuji IXGP each day for two weeks. Significant differences existed at all time points.

The developed FCR released large amounts of fluoride ions in the first three days, dropping rapidly thereafter (Figure 7a). The single-day release from the FCR on the fourth day was identical to that from the commercial control (Fuji IXGP), and the single-day release amount after four days was lower than that of the commercial control. These results also confirmed that Fuji IXGP exhibited fluoride release ability as claimed. However, the initial fluoride release rate and cumulative fluoride release (Figure 7b) of the commercial control were lower than those of the FCR.

#### 3.3.2. Fluoride Recharge Measurement

**Figure 8:**
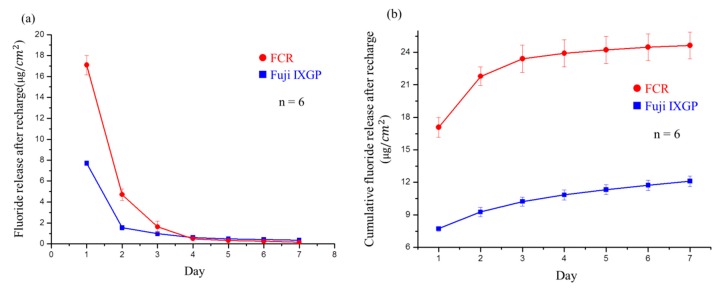
Analysis of fluoride release after recharge: (**a**) postrecharge fluoride release per cm^2^ of Fuji IXGP and the FCR each day for seven days. Significant differences were observed between the materials in the first three days; (**b**) cumulative fluoride release from Fuji IXGP and the FCR after recharge every day for seven days. Significant differences existed at all times.

As shown in Figure 8a, the developed FCR released a large amount of fluoride ions in the first two days after recharge with a 0.2% NaF solution. The FCR exhibited significantly higher cumulative fluoride ion release after recharge than Fuji IXGP (Figure 8b).

### 3.4. Biocompatibility (WST-1 Assay)

**Figure 9:**
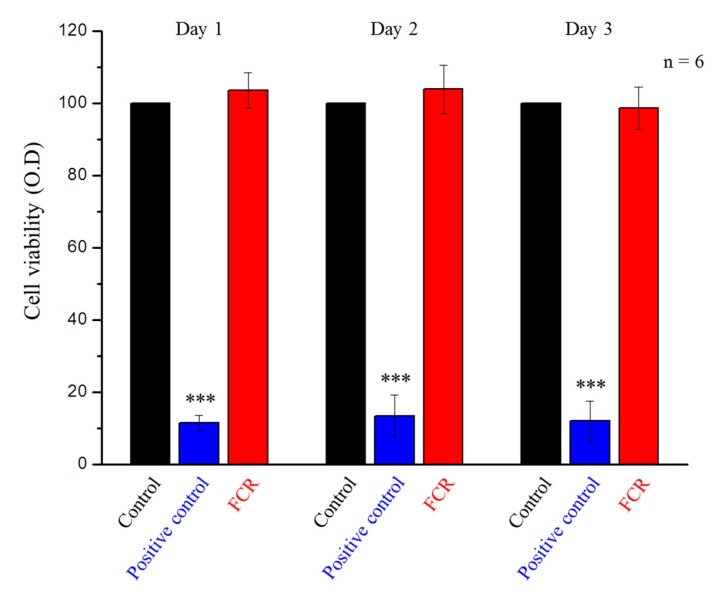
WST-1 assay of the control group, positive control group, and FCR extraction solution-treated 3T3 cells at days 1, 2, and 3. Only the positive control group was statistically significantly different from the other two groups (*** denotes *p* < 0.001).

There was no significant difference between the FCR group and the control group (Figure 9). These results confirmed that the FCR extraction solution did not cause cytotoxicity or inhibit cell growth in 3T3 cells.

## 4. Discussion

The aim of this study was to develop a new light-curing composite resin as a restorative material with good mechanical properties and fluoride release/recharge abilities. In the XRD analysis, the distance of the (001) layers was enlarged, after AAm was introduced into FMMT. The unmodified MMT diffraction angle was 5.58° and corresponded to a spacing of 1.58 nm; after a series of modifications, a plane spacing of 1.95 nm was calculated (converted from a diffraction angle of 4.63°). This expansion of the plane spacing was plausible, because the modified MMT contained a larger surface area to bind to fluoride ions and the interlayer polymer absorbed more NaF to allow for effective controlled release [40,41]. Furthermore, Figure 1b confirmed that the crystal structure of 3YSZ was trigonal but still included some monoclinic crystals. Additionally, the crystal structure of 3YSZ did not change after the reaction with MPTMS.

By FTIR analysis, we confirmed that the modified FMMT/AAm-NaF contained AAm [42], but it was difficult to determine whether the polymer was present on the surface of FMMT or intercalated into the layer structure. Therefore, TGA was used for verification (Figure 3a). Generally, the polymers will be immediately removed when heated to the boiling point, if the polymer is only present at the surface of an inorganic material. However, the thermogram showed that the weight percentage decreased in a stepwise manner with increasing temperature. This was due to the shielding effect of the smectic clay, which prevented the polymer inside the layers from being removed in a single step [43,44]. The FTIR spectroscopy revealed that the hydrophobically modified 3YSZ filler contained MPTMS (Figure 2b). Compared with pure MPTMS, the absorption bands at 1068 cm^−1^ (Si–O–Si) and 816 cm^−1^ (Si–O–C) of the 3YSZ/MPTMS became less pronounced, confirming that MPTMS were successfully coated on 3YSZ [45,46,47] and that the content was approximately 6.39 wt % (Figure 3b). The combined results of XRD, FTIR, and TGA confirmed that MMT and 3YSZ were successfully modified into hydrophobic fillers, which were effectively bonded with the polymer matrix.

Previous research indicated that particle size affects mechanical properties and surface smoothness of composite resins [26]. In this study, the FMMT/AAm-NaF average diameter obtained was ~1.25 μm, and the average diameter for the 3YSZ/MPTMS was ~0.73 μm. We believe that the inorganic fillers with this size can be distributed in the polymer matrix to provide suitable mechanical strength and smooth surface for the composite resin.

The hardening depth of the developed composite resin with the FMMT/AAm-NaF (20 wt %), 3YSZ/MPTMS (20 wt %), Bis-GMA matrix (60 wt %) was found to be significantly higher than that stated by the ISO 4049 standard [48]. The other mechanical properties were compared to those of Fuji IXGP, as this commercial product is the most commonly used when considering fluoride ion release. According to previous studies, mechanical properties of GICs are inferior to those of complex resins. The gel network formed by acid–base reactions in glass ionomers generally had lower strength and toughness than those for crosslinked polymers of Bis-GMA and TEGDMA in composite resins [49]. The results from Figure 6 showed that the composite resin developed in this study had high mechanical properties, which was in line with the expectations of this study. However, when compared with other commercial products with composite resin materials, the mechanical properties of our samples were significantly lower [50]. We speculated that this was due to a large amount of bubbles produced in the composite resin in the mixing process. Although we excluded samples that contained visible bubbles and burrs as much as possible, there may be imperfections in the samples that were not visible to the naked eye. We tried to remove the air from the samples by sonication or degassing using a Heidelberg hemisphere; however, it is possible that small air bubbles were not completely removed from the resin. These bubbles caused stress concentrations, which greatly reduced the mechanical properties of the FCR [51]. Thus, for FCR to be available as a reliable commercial product, air bubbles would have to be removed from the composite resin by manufacturers to obtain better mechanical properties.

To simulate the oral environment, we investigated the fluoride ions release and recharge ability of the FCR. The fluoride ion release was the highest in the first three days. We believe that, in addition to the fluoride ions released by FMMT, NaF in AAm released more fluoride ions. This can be explained by the fact that the concentration of fluoride ions released cannot reach such a high level after the recharge process. Based on previous studies, fluoride release rates of composite resins may be lower than those of GICs [52]. Therefore, we believe that NaF in water was absorbed by the FMMT on the surface of the composite resin in the recharge test. Interestingly, the amount released from the resin after recharge was still higher than that from the control product. Although Figure 8 showed that the amount of fluoride release rapidly declined, this is not concerning as patients typically use a fluoride-containing mouthwash or fluoride toothpaste approximately 2–3 times a day. This provides recharge opportunities to maintain fluoride ion concentrations from FCRs in the oral cavity.

A one-day FCR extraction was chosen for the WST-1 assay, because the FCR exhibited its highest fluoride ion release on the first day. We assumed that if the growth of 3T3 cells was not affected during the first day, additional days in contact with the extraction solution should not affect cell viability. Our data confirmed (Figure 9) that FCR materials were not toxic to cells and can therefore be used safely as a restorative dental material.

## 5. Conclusions

In this study, we used FMMT and hydrophobized 3YSZ as inorganic fillers in a Bis-GMA matrix to generate a composite resin with good fluoride ion release and recharge properties. The material analysis showed that these two different fillers were effectively modified and had good mechanical properties. In addition, in the oral environment simulation experiment, we compared the developed materials with commercial products that have a fluoride ion release effect in the clinic. The developed composite resin released higher concentrations of fluoride ions than GICs, making it a potential composite material that can replace GICs and be effectively used in the oral cavity to prevent secondary dental caries. In addition, the WST-1 biocompatibility assay showed that the FCR did not induce cytotoxicity. We believe that the FCR developed in this research study has the potential to be used in the clinical treatment of dental caries.
